# Editorial: Novel diagnostic and therapeutic strategies in the management of cerebral gliomas, Volume II

**DOI:** 10.3389/fonc.2023.1306332

**Published:** 2023-11-09

**Authors:** Alberto Bosio, Maria Caffo, Angelo Dipasquale, Marco Krengli, Matteo Simonelli, Giuseppe Lombardi

**Affiliations:** ^1^ Department of Oncology, Oncology 1 Veneto Institute of Oncology IOV-IRCCS, Padua, Italy; ^2^ Department of Biomorphology and Dental Sciences and Morphofunctional Imaging, Unit of Neurosurgery, University of Messina, Messina, Italy; ^3^ Department of Biomedical Sciences, Humanitas University, Milan, Italy; ^4^ Department of Medical Oncology and Hematology, IRCCS Humanitas Research Hospital, Milan, Italy; ^5^ DISCOG, University of Padua, Padua, Italy; ^6^ Division of Radiotherapy, Veneto Institute of Oncology, IOV-IRCCS, Padua, Italy

**Keywords:** glioma, novel therapeutic approach, biomarkers, immune system, immunotherapy

Gliomas are the most frequent primary central nervous system (CNS) tumors in adults, accounting for 81% of all intracranial malignancies (1). Based on 2021 World Health Organization (WHO) Classification of CNS, gliomas are classified into different entities depending on molecular profile and on histopathological characteristics (2). Despite multimodal treatment that is currently employed, including surgery, radiotherapy and chemotherapy, the patients’ prognosis still remains dismal. In recent years, the poor survival rate of these patients has led to major efforts to discover new and effective therapeutical strategies.

This Research Topic includes 4 manuscripts that highlight novel diagnostic and therapeutic approaches in the management of gliomas.

Ozone therapy is an established topic in medical care. Its efficacy is based on the ozone reactions and on the transient oxidative stress caused by its administration, that ultimately determine cancer growth inhibition. It has a variable toxicity profile depending on the concentrations with which it is administered, with allergic reactions and hemolysis being the most dangerous. It has been tested in different types of tumors, including colon, breast and lung cancer, with interesting results.

Based on the assumption that the glioma environment is mostly hypoxic with a predominance of aerobic glycolysis, a recent overview hypothesized that ozone therapy could be more effective for patients with glioma than for those with other tumors and that it may determine an improved sensitization to radiotherapy, one of the cornerstone of glioma therapy. Due to the contradictory outcomes reported in previous preclinical studies related to the different methods of administration, namely autohemotherapy or intratumoral injection of an oxygen/ozone mixture combined with concurrent STUPP protocol, further drug delivery studies are needed (Yanchu et al.).

With recent success of immunotherapy in different types of cancer, a growing interest of its application for gliomas patients has been observed. However, the initial enthusiasm has been tempered by the discovery that the glioma tumor microenvironment is highly immunosuppressive and heterogeneous, mostly dominated by the abundance of tumor-associated macrophages and low levels of infiltrating T-cells. Thus, delving into the mechanisms underlying the relationship between glioma and the immune system and investigating potential biomarkers, which can be useful tools in predicting which gliomas subtype may benefit most from immunotherapy, is needed. An additional potential perspective is represented by the combination of Immune checkpoint inhibitors and radiation that could enhance the sensitivity of tumor cells to immunotherapy.

Nicotinamide adenine dinucleotide (NAD+) metabolism represents one of the processes involved in cancerogenesis. A recent study (Jiang et al.) categorized glioma patients into two classes by profiling their NAD+ Metabolism Related Genes (NMRGS). The score derived from the expression level of the analyzed genes highlighted a NMRGS-high group and a NMRGS-low group. NMRGS score was found to directly correlate with expression level of immune checkpoint and with tumor mutational burden (TMB), suggesting that glioma patients with a higher NMRGS score may respond better to immune checkpoint inhibitors. In addition, survival analysis showed that patients with higher NMRGS score had poorer survival compared to those with lower NMRGS score, thus representing a possible future prognostic factor for patients with glioma.

A novel glioma biomarker relevant to the tumor microenvironment is Fas apoptosis inhibitory molecule 2 (FAIM2), a transmembrane protein involved in calcium homeostasis. It has been recognized to serve as an oncogene in several tumors, including non-small cell lung cancer, breast carcinoma and hepatocellular carcinoma. A recent study (Cai et al.) investigated the correlation between FAIM2 expression and the immune system in different types of cancer, with a particular focus on gliomas. It emerged that, unlike most of the other cancer types that have been taken into account, FAIM2 expression levels are inversely associated with immune infiltration, immunomodulators and immune checkpoint genes expression, tumor mutational burden (TMB), microsatellite instability (MSI) and mismatch repair (MMR) gene expression, suggesting that FAIM2 may be a potential immunotherapeutic target for gliomas. In addition, this results were strengthened by the demonstration that FAIM2 expression is downregulated in glioma tissues.

Zhou et al. reported that by analyzing expression data and clinical data from public databases (the Cancer Genome of Atlas (TCGA) and the Chinese Glioma Genome Atlas (CGGA), dual-specificity phosphatase 10 (DUSP10), an enzyme involved in cell proliferation and apoptosis, was found upregulated in gliomas when compared to normal brain tissue and significantly correlated with poor prognosis and clinical and molecular features, such as age, IDH mutation status and 1p/19q co-deletion. In addition, DUSP10 expression level was positively associated with glioma development, with immune infiltration and with the expression of immune checkpoints. Hence, targeting DUSP10 could be a novel therapeutic approach for glioma treatment.

In summary, the articles in this Research Topic presented interesting novel studies about new strategies in the management of gliomas, that need to be supported by prospective clinical trials in the future, and that enlighten and encourage us to further explore new experimental research to lead to an improved outcome for glioma patients.

## Author contributions

AB: Writing – original draft. MC: Writing – review & editing. AD: Writing – review & editing. MK: Writing – review & editing. MS: Writing – review & editing. GL: Writing – review & editing.
